# Chronic Administration of 13-*cis*-retinoic Acid Induces Depression-Like Behavior by Altering the Activity of Dentate Granule Cells

**DOI:** 10.1007/s13311-021-01168-6

**Published:** 2021-12-10

**Authors:** Xiao-Hong Su, Wei-Peng Li, Yi-Jie Wang, Jia Liu, Jun-Yu Liu, Ying Jiang, Fu-Hua Peng

**Affiliations:** 1 Department of Neurology, The Third Affiliated Hospital of Sun Yat-Sen University, Guangzhou, Guangdong People’s Republic of China; 2 State Key Laboratory of Organ Failure Research, Guangdong-Hong Kong-Macao Greater Bay Area Center for Brain Science and Brain-Inspired Intelligence, Guangdong Key Laboratory of Psychiatric Disorders, Collaborative Innovation Center for Brain Science, Department of Neurobiology, School of Basic Medical Sciences, Key Laboratory of Mental Health of the Ministry of Education, Southern Medical University, Guangzhou, Guangdong People’s Republic of China

**Keywords:** 13-*cis*-retinoic acid, Depression, Excitation/inhibition balance, Intrinsic membrane properties, Dentate gyrus

## Abstract

Depression is a common but serious mental disorder and can be caused by the side effects of medications. Evidence from abundant clinical case reports and experimental animal models has revealed the association between the classic anti-acne drug 13-*cis*-retinoic acid (13-*cis*-RA) and depressive symptoms. However, direct experimental evidence of this mechanism and information on appropriate therapeutic rescue strategies are lacking. Herein, our data revealed that chronic administration of 13-*cis*-RA to adolescent mice induced depression-like behavior but not anxiety-like behavior. We next demonstrated that chronic 13-*cis*-RA application increased neural activity in the dentate gyrus (DG) using c-Fos immunostaining, which may be critically involved in some aspects of depression-like behavior. Therefore, we assessed electrophysiological functions by obtaining whole-cell patch-clamp recordings of dentate granule cells (DGCs), which revealed that chronic 13-*cis*-RA treatment shifted the excitatory-inhibitory balance toward excitation and increased intrinsic excitability. Furthermore, a pharmacogenetic approach was performed to repeatedly silence DGCs, and this manipulation could rescue depression-like behavior in chronically 13-*cis*-RA-treated mice, suggesting DGCs as a potential cellular target for the direct alleviation of 13-*cis*-RA-induced depression.

**Supplementary Information:**

The online version contains supplementary material available at 10.1007/s13311-021-01168-6.

## Introduction

Depression is a serious mental disorder that is mainly characterized by anhedonia and behavioral despair and is associated with an increased risk of disability and mortality [1, 2]. Although the neurobiological mechanism is not fully understood in either primary or secondary depression, several recent studies have begun to identify aspects of neurobiological mechanisms underlying primary and secondary depression [1, 3, 4]. In contrast to primary depression, secondary depression can be caused by specific reasons, including organic diseases, psychotic disorders, medications, and so on [5–9]. It has been reported that treatment with the anti-acne drug Accutane (isotretinoin, 13-*cis*-retinoic acid [13-*cis*-RA]) has been associated with severe depression and even suicide [5, 10–13]. For all drugs, Accutane ranks fourth in the top five medications with the most frequent reports of depression [7]. Retinoic acid (RA) is the active metabolite of vitamin A (retinol) and includes the isoforms all-*trans* RA (ATRA), 9-*cis*-RA and 13-*cis*-RA. Furthermore, evidence from experimental animal models has also shown that RA is involved in the pathophysiology of depression. For example, chronic application of RA can induce hyperactivity of the hypothalamic–pituitary–adrenal (HPA) axis and behavioral changes especially depression-like behavior in rodents, and these behavioral changes can be attenuated by SN003, a corticotropin-releasing hormone receptor 1 antagonist [14–17]. In addition, Ro41-5253, a selective antagonist of RA receptor alpha (RARα), has been considered a potential novel strategy for the treatment of depression [18].

Emerging evidence has demonstrated that RA plays an important role in both the developing brain and the adult brain [10, 19–21]. RA signaling regulates many aspects of neuronal development, including synaptic plasticity, neurite outgrowth, and neurogenesis [19, 22–24]. Importantly, RA signaling has also been implicated in synaptic function and plasticity. Retinol deficiency leads to significantly impaired long-term potentiation (LTP) and long-term depression (LTD) in the mouse hippocampus, and this phenomenon can be reversed by reapplication of retinol [25]. In addition, RA, as a key mediator, has been implicated in regulating synaptic strength and plasticity [19, 24, 26, 27]. Recently, a study documented that intracerebroventricular (ICV) infusion of ATRA into the lateral ventricle of the rat brain leads to decreases in neuronal excitability and LTP in the hippocampus [28]. Despite extensive research, no current therapy exists for the rescue or prevention of RA-induced depression.

Given the role of 13-*cis*-RA in the pathophysiology of depression and synaptic plasticity, we set out to study the effects of chronic 13-*cis*-RA administration on the induction of depression-like behavior, as well as the changes in synaptic transmission and intrinsic excitability. Our data show that chronic administration of 13-*cis*-RA to adolescent mice induces depression-like behavior but not anxiety-like behavior and increases c-Fos expression levels in the dentate gyrus (DG), one of the classic emotion-related brain regions, suggesting a link between 13-*cis*-RA-induced depression and the alteration of synaptic transmission or intrinsic membrane properties of dentate granule cells (DGCs). We further demonstrate that 13-*cis*-RA treatment shifts the excitatory-inhibitory balance toward excitation and increases intrinsic excitability. Most importantly, we used a pharmacogenetic approach to decrease the neuronal activity of DGCs, and this manipulation could rescue depression-like behavior in chronically 13-*cis*-RA-treated mice. These results indicate that chronic 13-*cis*-RA treatment induces maladaptive synaptic and intrinsic plasticity, leading to hyperactivity of DGCs and subsequently causing depression-like behavior. Our findings suggest that DGCs in the DG are a key component of neural circuitry involved in mediating depression-like behavior and that decreasing DGCs neuronal activity could be a novel and effective treatment for 13-*cis*-RA-induced depression.

## Materials and Methods

### Mice

C57BL/6 male mice were obtained from the Sun Yat-sen University Animal Center (Guangzhou, China), which were within the adolescent age range for rodents (postnatal days 28–42). All mouse protocols were approved by the Institutional Animal Care and Use Committee at Sun Yat-sen University. Mice were housed in group cages (3–5 animals per cage) with free access to food and water under a controlled environment (20–24 °C, 45–55% humidity in a 12:12-h light/dark cycle). Mice were randomized into different treatment groups, and all experimental analyses were conducted by an investigator blinded to the treatment group.

### Drugs Administration

13-*Cis*-RA (Sigma, # R3255) was dissolved in darkness in corn oil with 10% ethanol and administered via intraperitoneal injection at a dose of 2 mg/kg/day for a period of 21 days. The control group received a mixture of ethanol and corn oil at a 1:9 ratio.

Clozapine-N-oxide (CNO) (Sigma, # SML2304) was dissolved in saline and freshly prepared before every experiment. The mice were intraperitoneally (ip) injected with saline or CNO (1 mg/kg) daily 20 min prior to treatment with 13-*cis*-RA. For electrophysiological verification of the effect of CNO in slices, CNO was diluted in artificial cerebrospinal fluid (ACSF) to a final concentration of 5 µM.

Doses and drug administration schedules were selected based on previous studies and pilot experiments in our laboratory.

### Behavioral Experiments

#### Sucrose Preference Test (SPT)

The SPT was performed to assess anhedonia [29]. Mice were trained to consume sucrose solution using a 24-h exposure period, in which two bottles of 1% sucrose solution were placed in each cage. After 24 h of food and water deprivation, the mice were given free access to two bottles (one with water and one with 1% sucrose) for 24 h, and the positions of the two bottles were switched every 6 h to reduce any side preference. The intake of sucrose solution and water was measured by weighing the bottles before and after the test. Sucrose preference was defined as the ratio of the weight of sucrose solution consumption to the weight of total fluid consumption.

#### Forced Swimming Test (FST)

The FST was performed to assess behavioral despair [30]. Briefly, mice were individually placed in a transparent glass cylinder (20 cm high, 20 cm diameter) containing water at a depth of 10 cm at 23–25 °C, and the immobility time was recorded during the last 4 min of the total 6-min trial.

#### Tail Suspension Test (TST)

TST was performed to assess behavioral despair [31]. Briefly, mice were suspended by their tails with adhesive tape, and the immobility time was recorded during a 6-min test session.

#### Open Field Test (OFT)

The OFT was performed to assess locomotor activity and was also suited for the evaluation of anxiety and neophobia. The OFT was performed in the open field apparatus with the VersaMax Animal Activity Monitoring System (Accuscan Instruments). Mice were gently placed in the center of an open field chamber (40 × 40 × 30 cm) and then allowed to freely explore for 30 min. The center 20 × 20 cm zone was defined as the central zone. The following parameters were automatically calculated using VersaDat software: the total distance traveled and the time spent in the central zone.

#### Light–Dark Transfer (LDT)

The LDT was performed to assess anxiety-like behavior. The LDT apparatus consisted of a rectangular Plexiglas box (42 × 21 × 25 cm) divided into a light compartment and a dark compartment of equal size, connected by a door. Mice were initially placed at the center of the light compartment and allowed to freely explore for 5 min. The percentage time spent in the light compartment and transition times between the two compartments were quantified.

#### Elevated Plus Maze (EPM)

The EPM was performed to assess anxiety-like behavior. The EPM consisted of two opposing open arms (30 × 5 × 0.5 cm) and two opposing enclosed arms (30 × 5 × 15 cm) that extended from a central platform (5 × 5 cm), which was 50 cm above the floor. Mice were placed in the central platform, facing one of the open arms, and recorded with an overhead camera as they freely explored the maze for 5 min. The following parameters were automatically calculated using video tracking software (Ethovision XT 11.5, Noldus): the distance traveled in the maze and the time spent in the open/closed arms.

#### Electrophysiological Recording

Hippocampal slices were prepared as described previously [32]. Briefly, mice were anesthetized with intraperitoneal injection of pentobarbital sodium (75 mg/kg) and then intracardially perfused with ice-cold oxygenated (95% O_2_ and 5% CO_2_) high-sucrose artificial cerebrospinal fluid (slice ACSF, in mM: 220 sucrose, 26 NaHCO_3_, 10 D-glucose, 12 MgSO_4_, 2 KCl, 1.3 NaH_2_PO_4_, and 0.2 CaCl_2_). The mice were sacrificed by decapitation, and their brains were rapidly removed and chilled in ice-cold oxygenated slice ACSF. Hippocampal areas were dissected using a vibratome (Leica, VT1200S), and the slices were immediately transferred to a holding chamber that contained oxygenated ACSF (recording ACSF, in mM: 124 NaCl, 26 NaHCO_3_, 10 D-glucose, 3 KCl, 1.25 NaH_2_PO_4_, 1 MgSO_4_ and 2 CaCl_2_, at pH 7.4, 305 mOsm) at 34 °C for 30 min and then maintained at room temperature for at least 1 h until required. A slice was transferred to the recording chamber on the stage of a microscope (Nikon, ECLIPSE FN1) with an infrared-sensitive camera (DAGE-MTI, IR-1000E), which was continuously perfused with oxygenated ACSF (2 ml/min) at 30 ± 1 °C. Recording pipettes were fabricated from filamented borosilicate glass capillaries and filament with a horizontal puller (Sutter Instruments, P-97). Recordings were undertaken using a multiclamp 700B amplifier and pClamp software (Molecular Devices). The data were acquired at 10 kHz and low-pass filtered at 2 kHz using Digidata 1550A (Molecular Devices).

Spontaneous excitatory postsynaptic currents (sEPSCs) were recorded in the presence of 20 µM bicuculline (BMI), and miniature EPSCs (mEPSCs) were recorded in the presence of 1 mM tetrodotoxin (TTX) + 20 µM BMI, while neurons were held in voltage-clamp mode at −70 mV. The pipette resistance was typically 4–6 MΩ filled with the internal solution (in mM: 130 K-gluconate, 20 KCl, 10 HEPES, 0.2 EGTA, 4 Mg-ATP, 0.3 Na-GTP, and 10 NaCreatine, at pH 7.3, 285 mOsm). Spontaneous inhibitory postsynaptic currents (sIPSCs) recordings were recorded in the presence of 1 mM kynurenic acid (KA), while neurons were held in voltage-clamp mode at 0 mV. The internal solution contained (in mM): 110 Cs_2_SO_4_, 0.5 CaCl_2_, 2 MgCl_2_, 5 EGTA, 5 HEPES, 5 TEA, and 5 Mg-ATP (pH 7.3, 285 mOsm). The paired-pulse ratio (PPR) of evoked EPSCs was conducted at −70 mV in the presence of 20 µM BMI with different interstimulus intervals and was calculated by the ratio of the amplitude of the second EPSC to that of the first EPSC. Intrinsic membrane properties, such as resting membrane potential (RMP), action potential (AP) threshold, input resistance (Rin), rheobase (minimal current required to induce neuronal firing), and firing number (APs induced by injecting sequentially increasing current steps), were recorded in current-clamp mode.

Cells were allowed to stabilize for approximately 2–5 min after break-in, and only recordings with stable series resistance were accepted (series resistance < 30 MΩ, change in series resistance < 20%, and absolute leak current < 100 pA). Data were analyzed using Minianalysis (Synaptosoft Inc.) and Clampfit 10.7 software (Molecular Devices).

#### Immunostaining

Mice were anesthetized with intraperitoneal injection of pentobarbital sodium (75 mg/kg) and then intracardially perfused with ice-cold saline, followed by 4% paraformaldehyde (PFA) in 0.1 M phosphate-buffered saline (PBS). The brains were removed, postfixed overnight at 4 °C in 4% PFA, and equilibrated in 30% sucrose in 0.1 M PBS. Coronal Sects. (40 µm) containing the regions of interest were cut on a cryostat (Leica CM, #1950); a series of coronal sections across the DG (every sixth section from bregma, −1.06 to −3.80 mm), paraventricular nucleus of the thalamus (PVT) (every sixth section from bregma, −0.94 to −2.06 mm), dorsal raphe nucleus (DRN) (every fourth section from bregma, −4.36 to −4.84 mm), medial prefrontal cortex (mPFC) (every fourth section from bregma, 1.98 to 1.7 mm), and nucleus accumbens (NAc) (every fourth section from bregma, 1.34 to 0.86 mm) were processed for staining. The sections were blocked with 5% bovine serum albumin in PBS containing 0.3% Triton, followed by incubation at 4 °C overnight with the following primary antibodies: c-Fos (1:500, Millipore, # ABE457) and NeuN (1:500, Cell Signaling Technology, # 24,307). The next day, the sections were washed and incubated with secondary antibodies (1:500, Invitrogen). Fluorescent images were captured using a confocal microscope (Nikon A1). For each region, the number of c-Fos-positive cells was counted using ImageJ software.

#### Stereotaxic Virus Injections

Mice were deeply anesthetized with 1% pentobarbital sodium (75 mg/kg) and placed in a stereotaxic frame (RWD Life Science). To target the DG, bilateral injections of adeno-associated virus (AAV) were made at two DG sites per hemisphere using the following coordinates (from bregma: mediolateral [ML] ± 1.70 mm, anteroposterior [AP] −2.00 mm, dorsoventral [DV] −2.00 mm and ML ± 1.85 mm, AP −2.92 mm, DV −2.00 mm). Viral solution was injected at a flow rate of 0.1 μl/min, and the glass pipette was withdrawn 5–10 min after the end of injection.

#### Real-Time Quantitative PCR

Total RNA was extracted from brain tissues using TRIzol reagent (Invitrogen, # 15596018), and reverse transcription was performed using the ReverTra Ace qPCR RT Kit (TOYOBO, # FSQ-101) according to the manufacturer’s instructions. The relative quantification of gene expression was performed using an ABI 7500 Real-Time PCR system (Applied Biosystems, USA) with SYBR Premix Ex Taq II (TaKaRa) under the following reaction conditions: 95 °C for 5 min, followed by 40 cycles at 95 °C for 20 s and 60 °C for 35 s.

The primers used for RT-PCR were as follows:

- Kir3.1-F: CAGCAGCTGGTGCCCAAGAAG
- Kir3.1-R: ACATGGGCTTTGTTCAGGTC
- Kir3.2-F: GTGAGGAAGGATGGGAAGTG
- Kir3.2-R: AGACAAACCCGTTGAGGTTG
- Kir3.3-F: TCGTAGTCATTCTCGAGGGC
- Kir3.3-R: CTGGGGATGGACCAGTAGAG
- Kcnf1-F: CGTGGCAGGCGAAGACATT
- Kcnf1-R: CCCCCGCCAAACAGTTGAT
- Kcnf2-F: ATGGGCAGTGTGAGAACCAAC
- Kcnf2-R: TGGACTTTACTCTTGCCATTCC
- Kcnn1-F: GCTCTTTTGCTCTGAAATGCC
- Kcnn1-R: CAGTCGTCGGCACCATTGTCC
- Kcnn2-F: GTCGCTGTATTCTTTAGCTCTG
- Kcnn2-R: ACGCTCATAAGTCATGGC
- Kcnn3-F: TGTTGCACTCTTCTCCCACG
- Kcnn3-R: GGTCATTGAGATTTAGCTGGCT

The relative quantity of mRNA was normalized to GAPDH (relative quantity = 2^−△△CT^).

### Statistical Analyses

Statistical analyses were carried out by the GraphPad Prism or SPSS software. Statistical differences among groups were analyzed using one-way analysis of variance (ANOVA), followed by Tukey’s post hoc test to evaluate differences between groups. Differences between two groups were analyzed by Student’s *t* test. All of the values were presented as mean ± s.e.m., and statistical significance was considered at *p* < 0.05.

## Results

### Chronic 13-*cis*-RA Treatment Induces Depression-Like Behavior

**Fig. 1:**
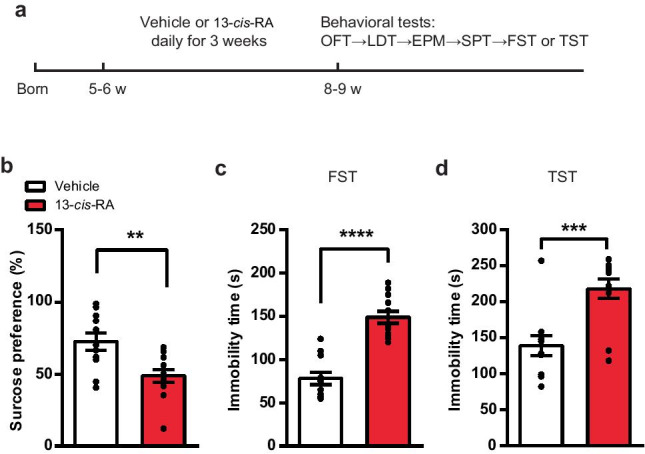
Chronic administration of 13-*cis*-RA induces depression-like behavior in mice. **a** Schematic representation of the experimental design. **b** Sucrose consumption in the SPT of chronically vehicle-treated and 13-*cis*-RA-treated mice (vehicle, *n* = 11 mice, 13-*cis*-RA, *n* = 12 mice; unpaired two-tailed Student’s *t* test, *t*_21_ = 3.244, *P* = 0.0039). **c** The immobility time in the FST of chronically vehicle-treated and 13-*cis*-RA-treated mice (vehicle, *n* = 11 mice, 13-*cis*-RA, *n* = 12 mice; unpaired two-tailed Student’s *t* test, *t*_21_ = 7.033, *P* < 0.0001). **d** The immobility time in the TST of chronically vehicle-treated and 13-*cis*-RA-treated mice (vehicle, *n* = 11 mice, 13-*cis*-RA, *n* = 12 mice; unpaired two-tailed Student’s *t* test, *t*_21_ = 4.099, *P* = 0.0005). The data are presented as the mean ± s.e.m. **P < 0.01, ***P < 0.001, ****P < 0.0001

To confirm whether the use of 13-*cis*-RA was associated with depression in adolescent mice, the SPT was performed to evaluate anhedonia, and the FST and TST were performed to evaluate behavioral despair (Fig. 1a). In the SPT, the sucrose consumption in the 13-*cis*-RA group was significantly decreased compared with that in the vehicle group (Fig. 1b). In the FST and TST, the immobility durations of 13-*cis*-RA-treated mice were significantly longer than those of the vehicle group (Fig. 1c, d). These results revealed that chronic administration of 13-*cis*-RA to adolescent mice induced depression-like behavior.

### Mice Treated with Chronic 13-*cis*-RA Do Not Show Anxiety-Like Behavior

**Fig. 2:**
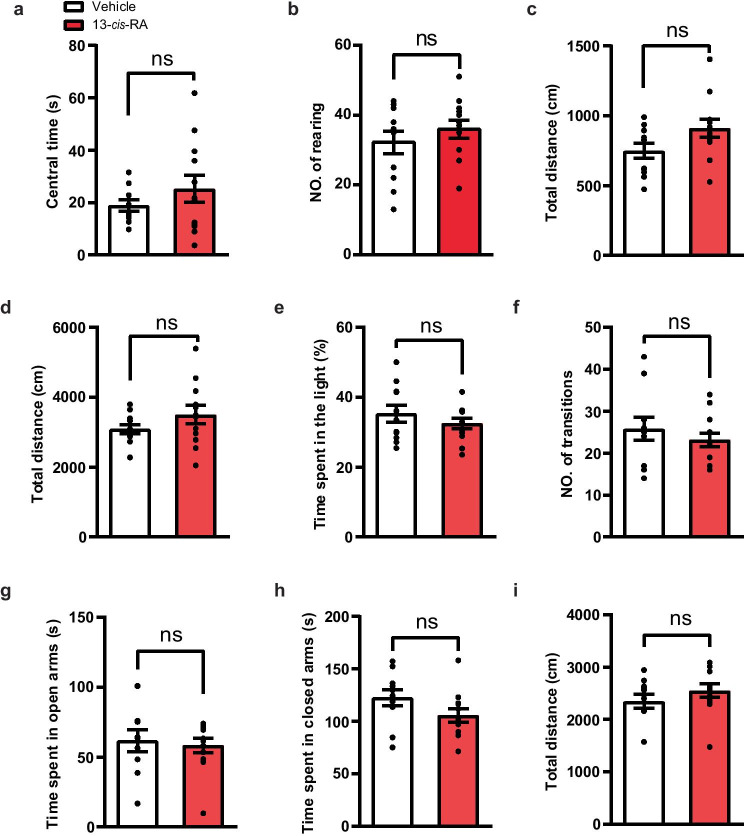
Chronic administration of 13-*cis*-RA does not induce anxiety-like behavior or abnormal locomotor activity in mice. **a** The time spent in the central zone (central zone duration) during the first 5 min of the OFT (vehicle, *n* = 11 mice, 13-*cis*-RA, *n* = 12 mice; unpaired two-tailed Student’s *t* test, *t*_21_ = 1.09, *P* = 0.2881). **b** The number of rearing (standing on the hind limbs) during the first 5 min of the OFT (vehicle, *n* = 11 mice, 13-*cis*-RA, *n* = 12 mice; unpaired two-tailed Student’s *t* test, *t*_21_ = 0.9108, *P* = 0.3727). **c** The distance moved during the first 5 min of the OFT (vehicle, *n* = 11 mice, 13-*cis*-RA, *n* = 12 mice; unpaired two-tailed Student’s *t* test, *t*_21_ = 1.892, *P* = 0.0723). **d** The distance moved in the 30 min of the OFT (vehicle, *n* = 11 mice, 13-*cis*-RA, *n* = 12 mice; unpaired two-tailed Student’s *t* test, *t*_21_ = 1.348, *P* = 0.192). **e** The time spent in the light chamber during the LDT (vehicle, *n* = 11 mice, 13-*cis*-RA, *n* = 12 mice; unpaired two-tailed Student’s *t* test, *t*_21_ = 0.9997, *P* = 0.3288). **f** The number of entries in the light chamber during the LDT (vehicle, *n* = 11 mice, 13-*cis*-RA, *n* = 12 mice; unpaired two-tailed Student’s *t* test, *t*_21_ = 0.8579, *P* = 0.4006). **g** The time spent in the open arms of the EPM (vehicle, *n* = 11 mice, 13-*cis*-RA, *n* = 12 mice; unpaired two-tailed Student’s *t* test, *t*_21_ = 0.3654, *P* = 0.7185). **h** The time spent in the closed arms of the EPM (vehicle, *n* = 11 mice, 13-*cis*-RA, *n* = 12 mice; unpaired two-tailed Student’s *t* test, *t*_21_ = 1.712, *P* = 0.1016). **i** The distance traveled in the EPM (vehicle, *n* = 11 mice, 13-*cis*-RA, *n* = 12 mice; unpaired two-tailed Student’s *t* test, *t*_21_ = 1.095, *P* = 0.286). The data are presented as the mean ± s.e.m. ns *P* > 0.05

It has been reported that depression is highly prevalent and often accompanied by comorbid anxiety disorder, suggesting that chronic 13-*cis*-RA may also induce anxiety-like behavior [1, 33, 34]. To address this question and to determine the effect of 13-*cis*-RA on anxiety-like behavior, we treated mice with 13-*cis*-RA or vehicle chronically and then evaluated anxiety-like behavior using the OFT, LDT, and EPM. In the OFT, we assessed the time spent in the center area as well as the number of rearing as indicators of anxiety. Our data revealed that 13-*cis*-RA-treated mice spent similar time in the central area and exhibited similar levels of rearing behavior during the first 5 min compared to those in the vehicle group (Fig. 2a, b). In the LDT, the time spent in the light chamber and the number of light chamber entries were not significantly different between the two groups (Fig. 2e, f). Similarly, in the EPM, no difference was observed in the time spent in the open and closed arms between the two groups (Fig. 2g, h). In addition, we did not observe significant differences in locomotor activities in the OFT and EPM (Fig. 2c, d, i). These results revealed that chronic administration of 13-*cis*-RA to adolescent mice did not induce anxiety-like behavior.

### Chronic 13-*cis*-RA Treatment Enhances Presynaptic Glutamate Release and Increases Intrinsic Excitability

**Fig. 3:**
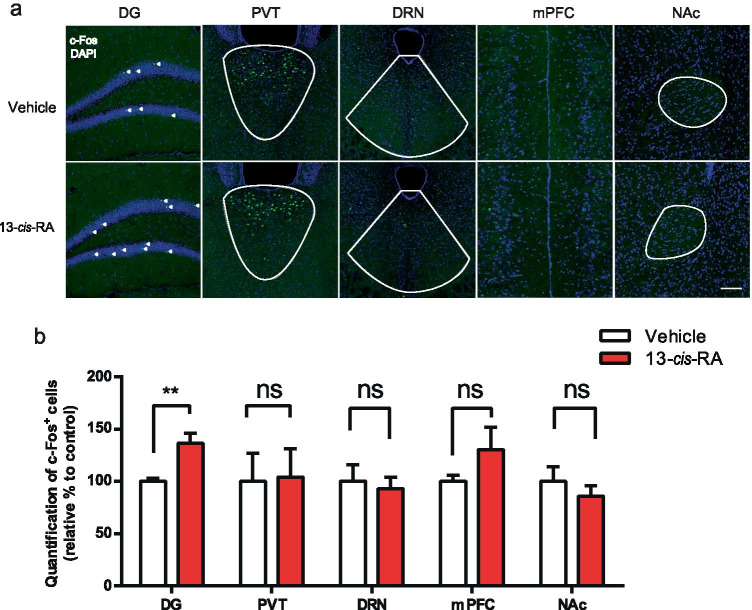
Administration of 13-*cis*-RA increased the expression of c-Fos in the DG of chronically 13-*cis*-RA-treated mice. **a** Representative images of c-Fos( +) expression in the DG, PVT, DRN, mPFC, and NAc. Scale bar, 100 μm. **b** Quantitative analysis of c-Fos( +) expression in the DG, PVT, DRN, mPFC, and NAc (*n* = 6 mice for each group; unpaired two-tailed Student’s *t* test, DG, *t*_10_ = 11.04, *P* = 0.0049; PVT, *t*_10_ = 0.1002, *P* = 0.9221; DRN, *t*_10_ = 0.3628, *P* = 0.7243; mPFC, *t*_10_ = 1.351, *P* = 0.2064; NAc, *t*_10_ = 0.8366, *P* = 0.4223). The data are presented as the mean ± s.e.m. **P < 0.01, ns *P* > 0.05

To further reveal the brain regions responding to depression-like behavior, we examined c-Fos expression levels by counting positive cells in brain regions relevant to depression-like behavior. As the expression of c-Fos was an indirect marker of neuronal activity, we monitored the effect of acute 13-*cis*-RA on c-Fos abundance, as an indication of increased neuronal activity, 30 min after intraperitoneal injection in chronic 13-*cis*-RA-treated mice. Our results showed that 13-*cis*-RA treatment increased c-Fos immunoreactivity in the DG but not in the PVT, DRN, mPFC, or NAc (Fig. 3).

**Fig. 4:**
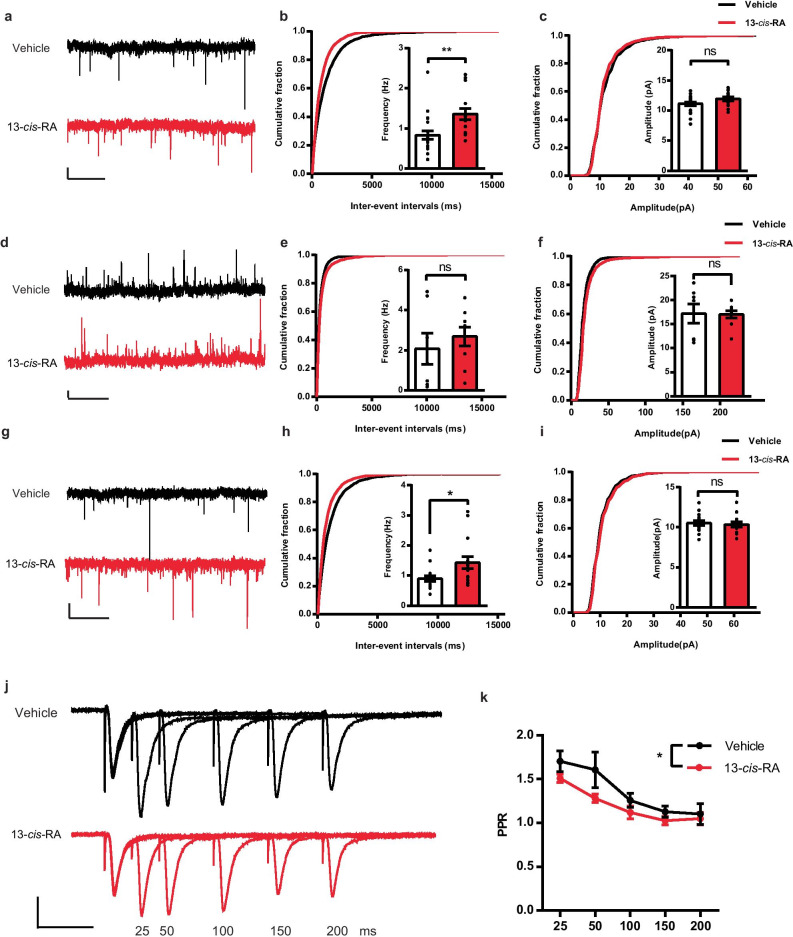
Excitatory synaptic input to DGCs is increased in chronically 13-*cis*-RA-treated mice. **a** Example traces of 10 s recordings of sEPSCs from DGCs in chronically vehicle-treated and 13-*cis*-RA-treated mice. Scale bars, 5 pA, 2 s. **b**–**c** Cumulative probability plots of the sEPSC interevent intervals (IEIs) and amplitude, with inserts depicting summary graphs of the frequency and amplitude of sEPSCs recorded from DGCs in chronically vehicle-treated and 13-*cis*-RA-treated mice, respectively (vehicle, *n* = 21 cells from 5 mice, 13-*cis*-RA, *n* = 15 cells from 4 mice; unpaired two-tailed Student’s *t* test, **b**, *t*_34_ = 3.096, *P* = 0.0039; **c**, *t*_34_ = 1.765, *P* = 0.0866). **d** Example traces of 10 s recordings of sIPSCs from DGCs in chronically vehicle-treated and 13-*cis*-RA-treated mice. Scale bars, 5 pA, 2 s. **e**–**f** Cumulative probability plots of the sIPSC IEIs and amplitude, with inserts depicting summary graphs of the frequency and amplitude of sIPSCs recorded from DGCs in chronically vehicle-treated and 13-*cis*-RA-treated mice, respectively (vehicle, *n* = 7 cells from 3 mice, 13-*cis*-RA, *n* = 9 cells from 3 mice; unpaired two-tailed Student’s *t* test, **e**, *t*_14_ = 0.7091, *P* = 0.4899; **f**, *t*_14_ = 0.08346, *P* = 0.9347). **g** Example traces of 10 s recordings of mEPSCs from DGCs in chronically vehicle-treated and 13-*cis*-RA-treated mice. Scale bars, 5 pA, 2 s. **h**–**i** Cumulative probability plots of the mEPSC IEIs and amplitude, with inserts depicting summary graphs of the frequency and amplitude of mEPSCs recorded from DGCs in chronically vehicle-treated and 13-*cis*-RA-treated mice, respectively (vehicle, *n* = 15 cells from 4 mice, 13-*cis*-RA, *n* = 15 cells from 4 mice; unpaired two-tailed Student’s *t* test, **h**, *t*_28_ = 2.379, *P* = 0.0244; **i**, *t*_28_ = 0.3609, *P* = 0.7209). **j** Example traces of PPR recorded from DGCs in chronically vehicle-treated and 13-*cis*-RA-treated mice. Scale bars, 100 pA, 50 ms. **k** Quantification of PPR recorded from DGC in chronically vehicle-treated and 13-*cis*-RA-treated mice (vehicle, *n* = 14 cells from 4 mice, 13-*cis*-RA, *n* = 12 cells from 3 mice; two-way ANOVA, *F*_(1, 120)_ = 6.057, *P* = 0.0153). The data are presented as the mean ± s.e.m. *P < 0.05, **P < 0.01, ns *P* > 0.05

However, the changes in c-Fos expression might be due to the alteration of synaptic transmission or intrinsic membrane properties in the DG. We next examined the changes in excitatory and inhibitory synaptic transmission, as well as the intrinsic membrane properties, using whole-cell patch-clamp recordings of DGCs in the DG of chronic 13-*cis*-RA-treated mice. Our results indicated that sEPSC frequency, but not amplitude, was significantly increased in the 13-*cis*-RA group compared with the vehicle group (Fig. 4a–c). However, we found no significant differences in sIPSC frequency or amplitude between the two groups (Fig. 4d–f). In addition, we then measured the changes in mEPSCs in the presence of TTX to block AP-driven synaptic activity. Our results showed that chronic administration of 13-*cis*-RA increased the mEPSC frequency without affecting the mEPSC amplitude, suggesting a presynaptic mechanism for the observed increase in glutamate-mediated synaptic transmission (Fig. 4g–i). Consistently, we found a decrease in the PPR in the 13-*cis*-RA group compared with that in the vehicle group (Fig. 4j, k). Taken together, the increase in mEPSC frequency in combination with reduction in the PPR strongly supported chronic 13-*cis*-RA-induced enhancement of presynaptic glutamate release, which reflected a shift of excitatory/inhibitory balance toward excitation.

**Fig. 5:**
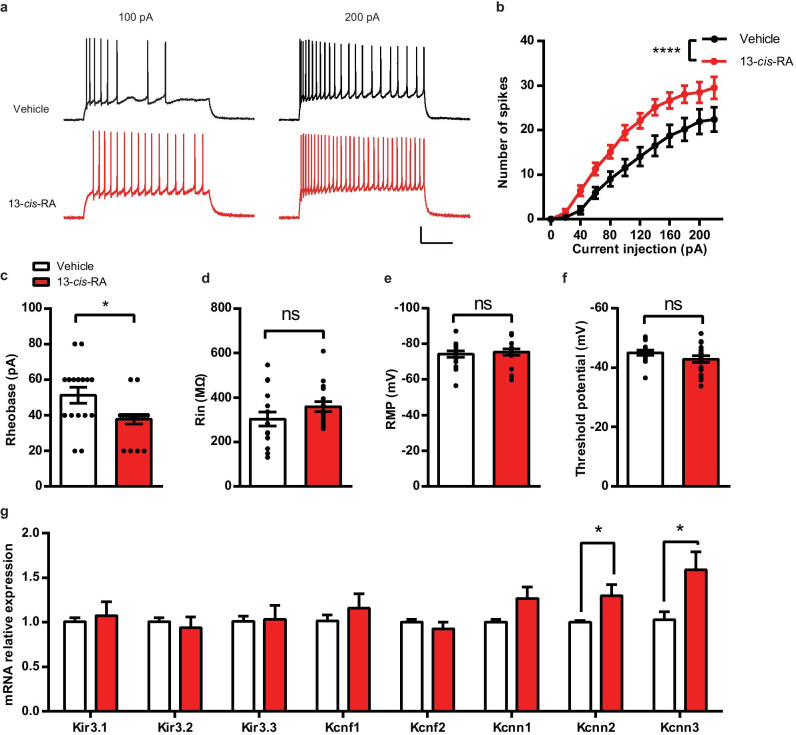
Effects of chronic administration of 13-*cis*-RA-induced modulation of neuronal excitability and membrane properties. **a** Representative traces of voltage responses (APs) elicited by depolarizing current steps of 100 and 200 pA in the DGCs of chronically vehicle-treated and 13-*cis*-RA-treated mice. Scale bars, 20 mV, 200 ms. **b**–**f** The parameters included the number of spikes elicited by incremental current steps, rheobase, Rin, RMP, and AP threshold potential in the DGCs of chronically vehicle-treated and 13-*cis*-RA-treated mice (vehicle, *n* = 18 cells from 5 mice, 13-*cis*-RA, *n* = 16 cells from 4 mice; two-way ANOVA, the number of spikes, *F*_(1, 384)_ = 61.58, *P* < 0.0001; unpaired two-tailed Student’s *t* test, rheobase, *t*_32_ = 2.635, *P* = 0.0129; Rin, *t*_32_ = 1.486, *P* = 0.1471; RMP, *t*_32_ = 0.4839, *P* = 0.6318; AP threshold potential, *t*_32_ = 1.475, *P* = 0.15). **g** RT-qPCR analysis of the indicated genes in the DG of chronically vehicle-treated and 13-*cis*-RA-treated mice (*n* = 8 mice for each group; unpaired two-tailed Student’s *t* test, Kcnn2, *t*_14_ = 2.373, *P* = 0.0325; Kcnn3, *t*_14_ = 2.511, *P* = 0.0249). The data are presented as the mean ± s.e.m. *P < 0.05, ****P < 0.0001, ns *P* > 0.05

To determine whether chronic administration of 13-*cis*-RA altered neuronal excitability, we next investigated intrinsic membrane properties, including the number of spikes elicited by incremental current steps, rheobase, Rin, RMP, and AP threshold potential. Our results showed that chronic administration of 13-*cis*-RA reduced rheobase and increased AP firing without altering RPM, AP threshold, and Rin compared to those in the vehicle group (Fig. 5a–f). In addition, to determine whether alterations in ion channel genes expression could potentially contribute to the hyperexcitability of DGCs, we performed RT-qPCR and found that the mRNA levels of Kcnn2 and Kcnn3 were significantly increased in chronic 13-*cis*-RA-treated mice (Fig. 5g).

Therefore, together with our observation of increasing excitatory/inhibitory balance, chronic administration of 13-*cis*-RA increased neuronal excitability, thereby enhancing neuronal activity in the DG.

### Decreasing the Activity of DGCs Through Inhibitory DREADDs Can Rescue Depression-Like Behavior in Chronically 13-*cis*-RA-Treated Mice

**Fig. 6:**
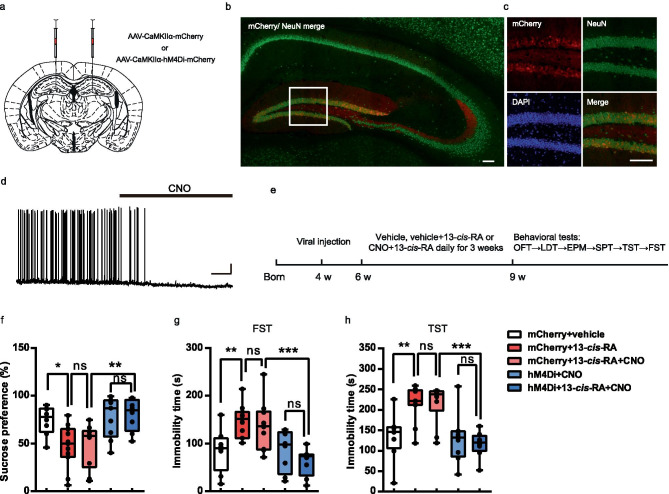
Decreasing the activity of DGCs through inhibitory DREADDs can rescue depression-like behavior in chronically 13-*cis*-RA-treated mice. **a** Schematic representation of stereotactic injection sites in the DG. **b**–**c** Representative images of the hippocampus/DG showing hM4Di-mCherry expression (red), NeuN expression (green), DAPI (blue), and the colocalization of hM4Di-mCherry and NeuN. Scale bar, 100 μm. **d** Representative trace recorded in current-clamp mode from a DGC that expressed hMD4i. Application of CNO (5 mM) induced hyperpolarization and abolished neuronal firing. Scale bar, 20 mV, 20 s. **e** Schematic representation of the experimental design. **f** The sucrose consumption in SPT (mCherry + vehicle, *n* = 8 mice, mCherry + 13-*cis*-RA, *n* = 11 mice, mCherry + 13-*cis*-RA + CNO, *n* = 10 mice, hM4Di + CNO, *n* = 8 mice, hM4Di + 13-*cis*-RA + CNO, *n* = 8 mice; one-way ANOVA, *F*_(4, 40)_ = 6.002, *P* = 0.0007). **g** The immobility time in the FST (mCherry + vehicle, *n* = 8 mice, mCherry + 13-*cis*-RA, *n* = 11 mice, mCherry + 13-*cis*-RA + CNO, *n* = 10 mice, hM4Di + CNO, *n* = 8 mice, hM4Di + 13-*cis*-RA + CNO, *n* = 8 mice; one-way ANOVA, *F*_(4, 40)_ = 7.224, *P* = 0.0002). **h** The immobility time in the TST. (mCherry + vehicle, *n* = 8 mice, mCherry + 13-*cis*-RA, *n* = 11 mice, mCherry + 13-*cis*-RA + CNO, *n* = 10 mice, hM4Di + CNO, *n* = 8 mice, hM4Di + 13-*cis*-RA + CNO, *n* = 8 mice; one-way ANOVA, *F*_(4, 40)_ = 9.096, *P* < 0.0001). The data are presented as the mean ± s.e.m. *P < 0.05, **P < 0.01, ***P < 0.001

If the increased neuronal activity of DGCs underlies the depression-like behavior observed in 13-*cis*-RA-treated mice, then silencing DGCs activity should ameliorate depression in these mice. To directly test this prediction, we first employed the chemogenetic designer receptors exclusively activated by designer drugs (DREADDs) approach to specifically express hM4Di-coupled designer receptors in DGCs. We bilaterally injected AAV-CaMKIIα-hM4Di-mCherry in the DG to express hM4Di specifically in DGCs. And AAV-CaMKIIα-mCherry was used as the control (Fig. 6a). Three weeks later, we confirmed AAV-mediated expression of hM4Di, which showed that mCherry fluorescence indicated the expression of the vector in the DG specially (Fig. 6b, c). To verify clozapine-N-oxide (CNO)-mediated inhibition of hM4Di-mCherry + DGCs, we performed whole-cell recordings in brain slices and found that application of CNO (5 mM) resulted in a significant decrease in firing activity (Fig. 6d). For chemogenetic manipulation, 2 weeks after the introduction of AAV-CaMKIIα-hM4Di-mCherry or AAV-hM4Di-mCherry, CNO (1 mg/kg) or vehicle was injected intraperitoneally daily 20 min prior to treatment with 13-*cis*-RA (Fig. 6e). Our results showed that sucrose consumption in the 13-*cis*-RA + mCherry group was significantly decreased compared to that in the vehicle + mCherry group, while CNO-mediated inhibition of hM4Di-mCherry + DGCs reversed 13-*cis*-RA-induced anhedonia (Fig. 6f). The immobility duration of 13-*cis*-RA-mCherry mice was significantly longer than that of the vehicle-mCherry group, while CNO-mediated inhibition of hM4Di-mCherry + DGCs reversed 13-*cis*-RA-induced behavioral despair (Fig. 6g, h). Furthermore, considering that CNO was converted to clozapine and that clozapine might be effective for depression and anxiety [35], another experimental group was set up, and our results showed that CNO application could not reverse 13-*cis*-RA-induced anhedonia and behavioral despair (Fig. 6g, h). In addition, silencing of DGCs with inhibitory DREADD did not induce anhedonia or behavioral despair (Fig. 6g, h). Meanwhile, no differences were observed in anxiety-like behavior or locomotor activity among the groups (Supplemental Fig. 1). Together, these results suggested that the activity of DGCs was critical for the depression-like behavior induced by chronic 13-*cis*-RA treatment, and targeting DGCs with inhibitory DREADDs could mitigate depression-like behavior.

## Discussion

In this study, we investigated the mechanism underlying 13-*cis*-RA-induced depression, focusing mainly on the role of electrophysiological alterations in DGCs, such as synaptic transmission and intrinsic membrane properties. Our study mimicked the effects of chronic clinical 13-*cis*-RA application in adolescents because acne is predominantly regarded as a skin disorder affecting adolescents [10, 36]. We found that 13-*cis*-RA treatment reliably induced depression-like behavior but not anxiety-like behavior. The increased neuronal activity in the DG was implied by elevated c-Fos expression levels in chronically 13-*cis*-RA-treated mice. Furthermore, the alteration of neuronal activity was assessed by obtaining whole-cell patch-clamp recordings of DGCs, and we found that chronic 13-*cis*-RA treatment shifted the excitatory-inhibitory balance toward excitation and increased intrinsic excitability. Silencing these neurons was sufficient to reverse 13-*cis*-RA-induced depression-like behavior. Thus, our findings indicated DGCs as a potential cellular target for the direct alleviation of RA-induced depression.

A significant body of evidence indicates that chronic 13-*cis*-RA administration is involved in both clinical depression and animal models of depression [5, 10–13, 37]. Clinically, 13-*cis*-RA is a highly effective drug for severe acne but is associated with depression and suicide, which has attracted major attention for a long time. In addition, animal model responses to 13-*cis*-RA administration support an association with psychiatric adverse effects, including depression and anxiety. Similar to previous studies, our data showed that chronic RA treatment reliably induced depression-like behavior. However, our results differed from those of previous studies reporting that chronic RA treatment also induced anxiety-like behavior [28]. Although depression and anxiety probably share some common neurophysiological mechanisms and behavioral manifestations, mechanisms associated with the behavioral appearances of depression and anxiety have not yet been completely understood [1, 33, 34]. The differences in these results are likely due to the differences in age period (i.e., adolescent vs. adult), species (i.e., mice vs. rats), and the method of administration (i.e., ip vs. ICV) or dose of the drugs.

RA is derived from retinol (vitamin A), which is abundantly expressed in the adult brain and plays a pivotal role in central nervous system development, including neurite outgrowth and synaptic plasticity. For example, our previous study highlighted the critical role of RA receptor gamma (RARγ) in modulating neurite outgrowth [23]. It has been reported that acute RA application can increase dendritic growth [38] and the expression of synaptic proteins in presynaptic and postsynaptic membranes, including synaptophysin and PSD-95, which are closely involved in synaptic plasticity [39]. RA can also regulate homeostatic synaptic plasticity through GluR1, an AMPA receptor subunit [40]. Furthermore, a growing body of studies indicates that RA enhances synaptic transmission, such as by increasing transmitter release in Xenopus cell culture or by increasing synaptic strength in neurons derived from human-induced pluripotent stem cells (iPSCs), with whole-cell patch-clamp recording [41, 42]. Interestingly, a recent study revealed that RA induces plasticity of excitatory synapses and dendritic spines in human cortical slices [27]. Our data indicate that chronic 13-*cis*-RA administration shifted the excitatory-inhibitory balance toward excitation, which is consistent with some of the previous experimental studies but is in conflict with the conclusion of a previous study [28]. One important difference between this study and our study is that they mainly focus on the extracellular field potentials in the hippocampal DG, which measure synaptically driven “field” population spikes (PS) or excitatory postsynaptic potentials (EPSP) evoked through a stimulating electrode. Therefore, it reflected a summation of postsynaptic potentials during simultaneous excitation of the DGCs and surrounding interneurons. Our study used whole-cell patch-clamp recordings to assess the effect of chronic administration of 13-*cis*-RA on the spontaneous synaptic transmission of individual DGCs. Furthermore, the differences in age, species, and the method of administration of the drugs should not be ignored, which might also contribute to different results. To our knowledge, changes in intrinsic membrane properties in DGCs have not been reported in a chronic 13-*cis*-RA-induced depression model, and these changes may be due to developmental defects such as neurogenesis in adolescent mice or other chronic compensatory changes in RA signaling. Interestingly, a recent study found that increased neuronal excitability of DGCs leads to depressive phenotypes [43], which is consistent with the electrophysiological features of DGCs observed in chronically 13-*cis*-RA-treated mice in our study. Altogether, these present results suggest a possible link between 13-*cis*-RA-induced depression and electrophysiological alterations in DGCs.

It is well known that the functional changes in DGCs are associated with the pathophysiology of depression and the beneficial effects of its most common treatments [44–47]. Since the results mentioned above suggested that increased neuronal activity in the DG may be a key player in the modulation of 13-*cis*-RA-induced depression-like behavior, we next examined the effect of silencing DGCs using a pharmacogenetic approach. Our results showed that this manipulation could rescue 13-*cis*-RA-induced depression, thereby providing experimental evidence to support the underlying mechanism of RA-induced depression and its therapeutic rescue. Thus, the findings of this study provide a potential cellular target for new therapeutic strategies, as well as new prospects and possibilities for the development of anti-acne drugs with more specific actions but fewer side effects.

## Supplementary Information

Below is the link to the electronic supplementary material.

Supplementary file1 (DOCX 226 KB)

Supplementary file2 (PDF 544 KB)

Supplementary file3 (PDF 552 KB)

Supplementary file4 (PDF 561 KB)

Supplementary file5 (PDF 570 KB)

Supplementary file6 (PDF 579 KB)

Supplementary file7 (PDF 588 KB)

Supplementary file8 (PDF 526 KB)
