# Supplementary material for: Chronic Administration of 13-cis-retinoic Acid Induces Depression-Like Behavior by Altering the Activity of Dentate Granule Cells
Source: Neurotherapeutics. 2021 Dec 10;19(1):421–33. doi: 10.1007/s13311-021-01168-6 (PMC9130401; doi:10.1007/s13311-021-01168-6)
Supplement: Supplementary file 1 — Supplementary file1 (DOCX 226 KB) [file 13311_2021_1168_MOESM1_ESM.docx]

**Supplementary Information**


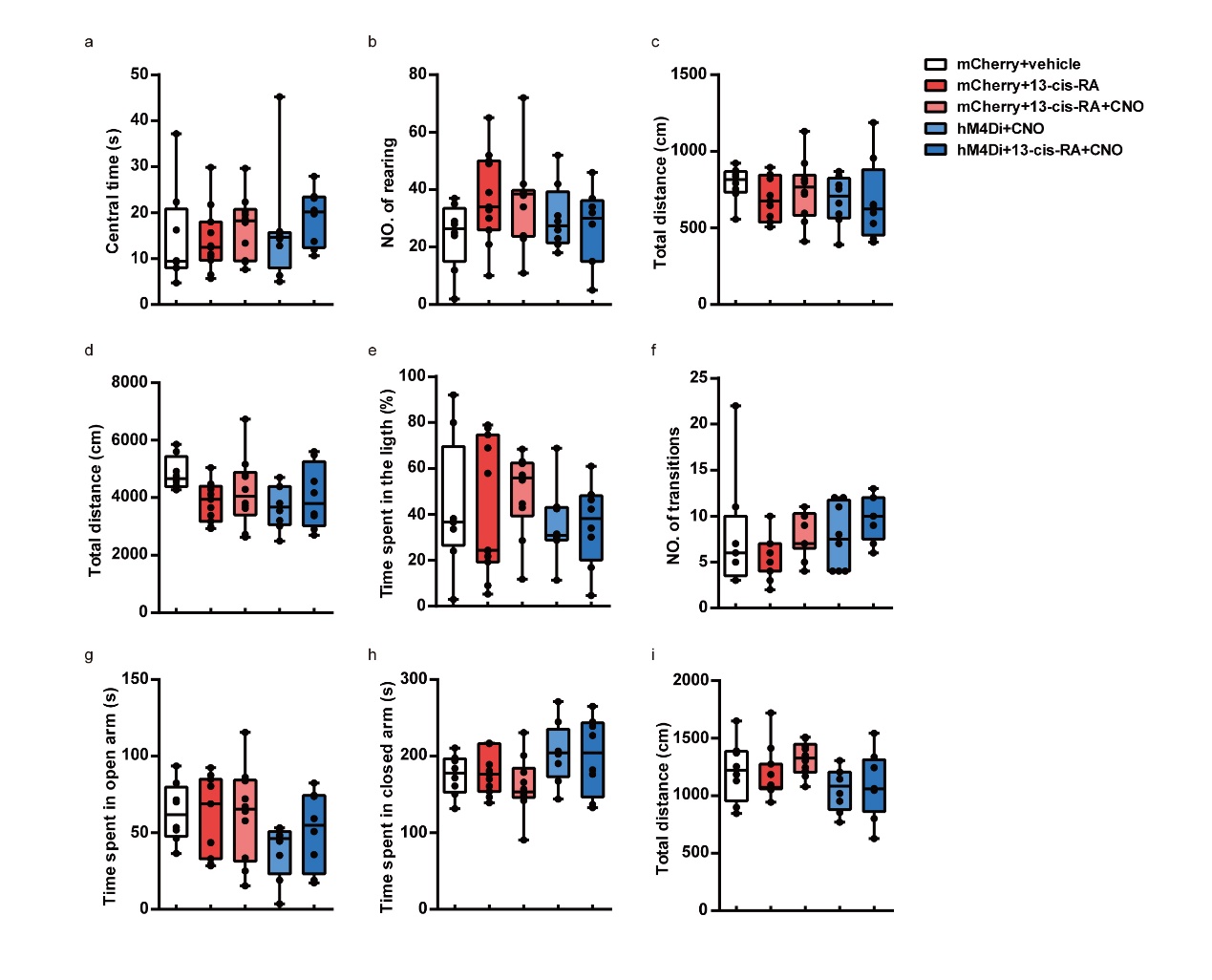


**Supplemental Fig. 1 Decreasing the activity of DGCs through inhibitory DREADDs does not induce anxiety-like behavior or abnormal locomotor activity in mice.**

a. The time spent in the central zone (central zone duration) during the first 5 min of the OFT. (mCherry+vehicle, n=8 mice, mCherry+13-*cis*-RA, n=11 mice, mCherry+13-*cis*-RA+CNO, n=10 mice, hM4Di+CNO, n=8 mice, hM4Di+13-*cis*-RA+CNO, n=8 mice; One-way ANOVA, F _(4, 40)_ = 0.4499, P = 0.7718).

b. The number of rearing (standing on the hind limbs) during the first 5 min of the OFT. (mCherry+vehicle, n=8 mice, mCherry+13-*cis-*RA, n=11 mice, mCherry+13*-cis-*RA+CNO, n=10 mice, hM4Di+CNO, n=8 mice, hM4Di+13*-cis-*RA+CNO, n=8 mice; One-way ANOVA, F _(4, 40)_ = 1.537, P = 0.2099).

c. The distance moved during the first 5 min of the OFT. (mCherry+vehicle, n=8 mice, mCherry+13*-cis-*RA, n=11 mice, mCherry+13*-cis-*RA+CNO, n=10 mice, hM4Di+CNO, n=8 mice, hM4Di+13*-cis-*RA+CNO, n=8 mice; One-way ANOVA, F _(4, 40)_ = 0.6249, P = 0.6474).

d. The distance moved during the 30-min duration of the OFT. (mCherry+vehicle, n=8 mice, mCherry+13*-cis-*RA, n=11 mice, mCherry+13*-cis-*RA+CNO, n=10 mice, hM4Di+CNO, n=8 mice, hM4Di+13*-cis-*RA+CNO, n=8 mice; One-way ANOVA, F _(4, 40)_ = 1.995, P = 0.1138).

e. The time spent in the light chamber during the LDT. (mCherry+vehicle, n=8 mice, mCherry+13*-cis-*RA, n=11 mice, mCherry+13*-cis-*RA+CNO, n=10 mice, hM4Di+CNO, n=8 mice, hM4Di+13*-cis-*RA+CNO, n=8 mice; One-way ANOVA, F _(4, 40)_ = 0.5325, P = 0.7125).

f. The number of entries in the light chamber during the LDT. (mCherry+vehicle, n=8 mice, mCherry+13*-cis-*RA, n=11 mice, mCherry+13*-cis-*RA+CNO, n=10 mice, hM4Di+CNO, n=8 mice, hM4Di+13*-cis-*RA+CNO, n=8 mice; One-way ANOVA, F _(4, 40)_ = 2.196, P = 0.0868).

g. The time spent in the open arms of the EPM. (mCherry+vehicle, n=8 mice, mCherry+13*-cis-*RA, n=11 mice, mCherry+13*-cis-*RA+CNO, n=10 mice, hM4Di+CNO, n=8 mice, hM4Di+13*-cis-*RA+CNO, n=8 mice; One-way ANOVA, F _(4, 40)_ = 1.555, P = 0.2051).

h. The time spent in the closed arms of the EPM. (mCherry+vehicle, n=8 mice, mCherry+13*-cis-*RA, n=11 mice, mCherry+13*-cis-*RA+CNO, n=10 mice, hM4Di+CNO, n=8 mice, hM4Di+13*-cis-*RA+CNO, n=8 mice; One-way ANOVA, F _(4, 40)_ = 2.091, P = 0.1001).

i. The distance traveled in the EPM. (mCherry+vehicle, n=8 mice, mCherry+13*-cis-*RA, n=11 mice, mCherry+13*-cis-*RA+CNO, n=10 mice, hM4Di+CNO, n=8 mice, hM4Di+13*-cis-*RA+CNO, n=8 mice; One-way ANOVA, F _(4, 40)_ = 1.957, P = 0.1197).

The data are presented as the mean ± s.e.m. ns P > 0.05.
